# Quantification and diagnostic relevance of blood and heme-mediated inhibition of prion detection by RT-QuIC

**DOI:** 10.1128/jcm.00615-25

**Published:** 2025-12-18

**Authors:** Robert B. Piel, David A. Schneider

**Affiliations:** 1 Department of Microbiology and Pathology, Washington State University 6760, Pullman, Washington, USA; 2 Animal Disease Research Unit, United States Department of Agriculture, Agricultural Research Service, Pullman, Washington, USA; University of California, Davis, Davis, California, USA

**Keywords:** hemoglobin, heme, blood, real-time quaking-induced conversion, prions

## Abstract

Prion diseases are characterized by misfolding of prion protein (PrP) from correctly folded PrP^C^ to a disease-associated form, PrP^D^. Real-time quaking-induced conversion (RT-QuIC) detects prions by “seeding” reaction mixtures, which contain recombinant PrP, with samples suspected to contain prions, resulting in amplification of misfolded PrP. The assay is sensitive to inhibition by tissue constituents, including blood. Heme, a cofactor of hemoglobin (Hb), has been shown to bind PrP in an isoform-specific manner and to affect the stability of other pathogenic amyloids. Herein, tissue samples from scrapie-positive sheep were used to seed RT-QuIC reactions in the presence of heme—as free hemin, as a cofactor of Hb, and as present in whole blood. At equivalent heme concentrations, the inhibitory action of free heme was the least and that of blood the greatest, suggesting other components of Hb and whole blood have additional inhibitory actions. We also demonstrate that this inhibition of RT-QuIC acts through disruption of the recombinant PrP assay substrate, rather than destruction of PrP^D^ seeds. Lastly, heme concentrations were measured in several ruminant tissues. Heme levels exceeded inhibitory thresholds in nearly all types of intact tissue but were reduced below inhibitory levels at a 1:1,000 dilution of most tissue types, with whole blood being one of a few notable exceptions. Our results suggest that detection of PrP^D^ seeding activity is not precluded by exposure to heme in tissue samples, but that the final heme concentration introduced into the RT-QuIC assay mixture is the critical factor that impacts detection sensitivity.

**IMPORTANCE:**

Real-time quaking-induced conversion (RT-QuIC) is an ultrasensitive amplification assay for the detection of prions. The assay has shown exceptional performance in optimal laboratory conditions, on par with bioassay, and far surpassing current immunoassay diagnostics. However, efforts to apply RT-QuIC as a real-world diagnostic have been hampered by inconsistencies and unexpectedly low sensitivity in some field samples. This study aims to quantify and characterize the mechanism of inhibition from blood and its constituent parts, hemoglobin and heme—omnipresent components of most sample types. Such systematic evaluations of RT-QuIC inhibitory factors represent necessary steps toward the consistent and sensitive performance necessary for a field-applicable diagnostic assay.

## INTRODUCTION

In prion diseases, which include scrapie in sheep and goats, chronic wasting disease (CWD) in cervids, and various others in both agricultural animals and humans, initial transmission is followed by a prolonged asymptomatic incubation period, which can often last years. As infectious prions are shed during preclinical infection (1, 2), these animals represent a critical management challenge to efforts targeting highly transmissible prion diseases. These include the final stages of eradication for classical scrapie in the USA as well as responses to the continuing expansion of CWD across North America. Currently, the diagnosis of prion disease in small ruminants and cervid species is accomplished by immunoassay using lymphoid tissues typically collected postmortem. A reliable and highly sensitive assay for early-stage prion disease using peripheral tissues or blood would greatly aid in disease management efforts.

A defining feature of prion infection is the replication of the infectious unit (the prion) by templated misfolding of natively expressed prion protein (PrP) from its normal “cellular” form (PrP^C^) to a misfolded “disease” form (PrP^D^) (3). Amplification assays such as real-time quaking-induced conversion (RT-QuIC) exploit this inherent mechanism of replication to enable detection of minuscule amounts of misfolded protein that may otherwise elude conventional methods such as immunoassay. In RT-QuIC, enhanced detection is accomplished by exposing a reaction mixture containing monomeric, metastable recombinant prion protein (rPrP) substrate to test samples that may contain prions (4, 5). In the case of a positive sample, PrP^D^ in the sample acts as a template or “seed” for the conversion of the rPrP substrate to a misfolded form. The assay then proceeds in cycles of shaking, through which the newly misfolded substrate can also participate in seeding further conversion, thereby amplifying the amount of misfolded prion protein in the reaction mixture to detectable levels.

The RT-QuIC assay has shown exceptional sensitivity for the detection of prions, where seeding activity can be detected in positive samples that have been diluted more than 10 billion-fold from the starting tissue (5). However, a significant hurdle facing the assay is that RT-QuIC is inhibited by various constituents naturally present in tissues and bodily fluids. A common practice has been to dilute tissue samples 1,000-fold (10^−3^) before use in the assay (5–7). While this technique does succeed in circumventing a large part of tissue-mediated inhibition, it also inherently limits the potential sensitivity of the assay, thereby limiting the potential of amplification assays to enhance the detection of early infections.

In addition to “tissue” generally, constituents of blood and, in particular, red blood cells are known to strongly inhibit the RT-QuIC assay (8–11). This is problematic both because blood is a near-ubiquitous contaminant of most tissues and because blood itself is known to harbor infectious prions (12–16) and is thus a very attractive sample type for live animal testing that could be repeated over time. While some progress has been made in detecting bloodborne prions by RT-QuIC with pre-analytic processing techniques such as selective precipitation (12) and affinity purification (10), it is clear that some level of inhibition is still present relative to non-blood-exposed samples (11). In contrast to other cell types, red blood cells, or erythrocytes, are unique in that >95% of their dry mass is made up of hemoglobin (Hb)(17, 18). Critical to its oxygen-carrying function, each tetrameric Hb protein contains four heme molecules as cofactors. Free heme (hemin in its oxidized form) is a highly reactive porphyrin molecule that is known to cause oxidative damage (19, 20) when its normal cellular niche is disrupted, such as after hemolysis or ischemia-reperfusion injury, conditions that are variably present during tissue sampling, storage, and homogenization. In addition to its potential to cause oxidative damage, heme also binds to PrP in an isoform-sensitive manner (21–23). While physiologic functions of PrP^C^-heme binding have been proposed (21, 22), the implications for misfolding assays are not entirely clear. In addition to erythrocyte-mediated inhibition of RT-QuIC (8), hemin itself has previously been shown to inhibit the detection of prions by another type of misfolding assay, the protein misfolding cyclic amplification (PMCA) assay (24). Furthermore, hemin has also been shown to influence the structures and stability of other disease-associated amyloids, including amyloid-β of Alzheimer’s disease (25, 26), α-synuclein of Parkinson’s disease (27), and lysozyme of lysozyme amyloidosis (28).

The present study aims to elucidate the extent to which, and processes by which, blood and its major components, hemoglobin and heme, inhibit the RT-QuIC assay in the sensitive detection of prions in tissue samples. This knowledge may ultimately allow for mitigation of this inhibition and the more sensitive detection of prions in blood-containing samples and/or blood directly.

## MATERIALS AND METHODS

### Tissue preparation

The tissue samples used in this study are from a frozen archive of tissues collected from naturally infected and experimentally infected small ruminants. All tissue samples were collected after humane euthanasia and were same-day processed and frozen (−80°) until use.

Sheep brain homogenates for RT-QuIC seeding (#4789 [pos], #4799 [neg]) were prepared as 10% (wt/vol) homogenates in 1× phosphate-buffered saline (PBS) using a rotor stator homogenizer (GLH-01, Omni International) with single-use plastic probe tips.

Blood samples for RT-QuIC inhibition and rPrP binding studies were collected from a scrapie-naïve sheep (#5047) in 10 mL EDTA Vacutainer tubes (Becton Dickinson). Tissue samples for heme quantification were collected from two sheep (#4645, #4649) and one goat (#5010G). Tissues collected included brainstem, cerebellum, tonsil, retropharyngeal lymph node (RPLN), spleen, kidney, liver, diaphragm, skeletal muscle, rectal mucosa, cerebrospinal fluid (CSF), and whole blood collected with ACD anticoagulant (8:60 mL ACD Formula A; Fenwal). Sheep placental cotyledons were also collected, with five individual cotyledons gathered from each of 10 placentas (#1266–1275). Tissues for heme quantification experiments, excluding blood and CSF, were prepared as 10% (wt/vol) homogenates in 1× PBS using 0.7 mm Zirconia beads (BioSpec 11079107zx) in a bead-beating grinder (Fast Prep 24; MP bio). CSF was prepared by centrifugation at 500 × *g* for 10 min, and the resulting supernatant was collected. Tissue homogenate, CSF supernatant, and whole blood stocks were stored at −80°C, with working sub-aliquots stored short term at −20°C.

Homogenate concentrations in this manuscript are described as dilutions relative to intact tissues, i.e., a 10% (wt/vol) homogenate is represented as a 10^−1^ dilution.

### Heme and Hb solution preparation

Hemin (Hemin-Cl; EMD Millipore) and Hb (Hemoglobin from bovine blood; Sigma Aldrich) stock solutions were prepared gravimetrically from dry reagents.

Hemin was solubilized in 0.1 M NaOH and subsequently diluted at least 100-fold in seed dilution (SD) buffer (1× PBS pH 7.4 + 0.1% SDS + 1× N-2 media supplement [Gibco-Fisher]) to amend pH; hemin was found to be soluble in this buffer to at least 200 µM.

Hb was prepared in 1× PBS pH 7.4 for quantification standards or in SD buffer for RT-QuIC spiking. For Hb, concentrations are calculated and reported as monomeric Hb.

Apohemoglobin (apoHb) was prepared from Hb stock solutions by acid-acetone extraction (29, 30). A solution of 20 mM Hb was added dropwise with constant stirring to approximately 30 volumes of acidified acetone (0.2% vol/vol 12 N HCl) at −20°C. The resulting precipitate was collected by centrifugation at 1,000 × *g* for 15 min and resuspended in ddH_2_O. The resulting solution was then successively dialyzed against ddH_2_O, 1.6 mM sodium bicarbonate, and 1× PBS pH 7.4. Following dialysis, residual precipitate was removed by centrifugation at 3,000 × *g* for 10 min. ApoHb concentrations were estimated by UV-Vis absorbance at 280 nm using ε_280nm_ = 0.0162 M^−1^ (29, 31). ApoHb dilutions prepared using this extinction coefficient were also compared to (wt/vol) holohemoglobin (holoHb) standards by SDS-PAGE and Coomassie staining.

### Heme quantification in blood and tissues

Heme concentrations in whole blood were measured by alkaline detergent hematin (ADH) assay (32, 33). Dilutions of whole blood and Hb standards were prepared in 1× PBS. A total of 20 µL of each was then added to 180 µL of a buffer consisting of 0.1 M NaOH + 2.5% wt/vol Triton X-100. UV-Vis absorbance spectra were recorded, and blood-heme concentrations were calculated by comparison to the Hb standard curve at 575 nm.

Heme concentrations in sheep and goat tissues were measured by oxalic acid fluorescence assay (34, 35). Dilutions of tissue homogenates and Hb standards were prepared in 1× PBS. A total of 20 µL of each was then added to 980 µL of 2 M oxalic acid. The sample preparations were then split, and 500 µL was incubated at 100°C for 30 min while the other half was maintained at room temperature. Samples were then measured for fluorescence using an excitation wavelength of 400 nm and emission of 662 nm. For each sample, the room temperature measurements were subtracted from the 100°C incubated measurements to control for any non-heme-derived fluorescence present in the samples. Measurements from the Hb standard curve were then used to calculate the concentration of heme for tissue samples.

UV-Vis and fluorescence measurements were performed using a CLARIOstar microplate reader (BMG Labtech).

### RT-QuIC

RT-QuIC was performed using hamster-sheep chimeric rPrP substrate (Syrian hamster residues 23 to 137 [accession no. K02234] followed by sheep [R154, Q171] residues 141 to 234 [accession no. AJ567988]). Protein was expressed in DE3 *Escherichia coli* using the pET41 vector and Overnight Express Autoinduction System 1 (Novagen, Madison, WI). rPrP was purified from inclusion bodies as described by Orrú et al. (4). Briefly, inclusion bodies were solubilized in guanidine hydrochloride, purified using nickel immobilized metal affinity chromatography, and refolded on the resin with a gradient of guanidine hydrochloride using an AKTA Pure FPLC (Cytivia). Following elution by imidazole gradient, rPrP was dialyzed into 10 mM Na_2_PO_4_ (pH 5.8) and stored at −80°C.

RT-QuIC reactions, consisting of 2 µL seed material and 98 µL RT-QuIC assay buffer (10 mM NaPO_4_ pH 7.4, 300 mM NaCl, 1 mM EDTA, 10 µM ThT, 0.1 mg/mL rPrP) per well, were carried out at 42°C with alternating cycles of 1 min double orbital shaking at 700 rpm and 1 min rest for 100 h total using FLUOstar or CLARIOstar microplate readers (BMG Labtech). ThT fluorescence was measured using 20 flashes per well, bottom read, with an excitation wavelength of 450 ± 10 nm and emission wavelength of 480 ± 10 nm, fixed gain of 1,800, and 15 min read intervals.

For RT-QuIC, seed homogenate dilutions were prepared in SD buffer (1× PBS pH 7.4 + 0.1% SDS + 1 × N-2 media supplement [Gibco-Fisher]). In RT-QuIC reactions containing hemin, Hb, or blood, inhibitors were spiked into the seed homogenate dilutions such that the concentrations named represent the final concentrations present in the seed material. For blood, reported concentrations represent the heme/monomeric Hb concentration present in each dilution of whole blood.

### Methods used to evaluate RT-QuIC data

Each 100 h record of ThT fluorescence measurements was exported to an Excel spreadsheet. A custom script (Python version 3.10.9) was written to import, merge, and export as one comma-delimited data file all Excel files relevant to a given experiment. Each experiment’s data file was then imported into SAS (SAS version 9.4), where various SAS procedures (PROCs) were applied to transform data, detect and characterize reactions, perform statistical analyses, and produce graphs for presentation.

RT-QuIC reaction data, including Excel exports of raw ThT fluorescence measurements and graphs depicting raw ThT fluorescence curves, are available from the National Agricultural Library Ag Data Commons database (https://doi.org/10.15482/USDA.ADC/28836296). Examples of raw ThT fluorescence curves can be seen in Fig. 8A of this manuscript.

Given some extreme effects of the conditions of this study on the morphology and variability in the fluorescence data, in place of a traditional plate-wide fluorescence threshold, custom algorithms were created and uniformly applied to each individual well’s data to detect reactions and evaluate morphologic features. The fluorescence readings were first regressed over time (PROC TRANSEG) to produce its penalized B-spline and its upper 99.9% confidence limit. This resulted in the local reduction in variability and a liberal upper confidence limit. Subsequently, a moving estimate of the fluorescence trend (ThT_trend_) was calculated as the leading 1 h median of the penalized B-spline (PROC EXPAND). Similarly, a moving estimate of a critical threshold value (ThT_crit_) was calculated as the preceding 5 h median of the fluorescence upper confidence limit offset by an additional 30 min. Thus, at a given time point, a relatively short forward-biased estimate of ThT_trend_ was compared to a local window estimate of the preceding baseline fluorescence (ThT_crit_). A positive amplification signal and the accompanying lag time for a given reaction was determined by whether and when ThT_trend_ first exceeded ThT_crit_. The reaction height at a given time point was defined as the baseline (ThT_crit_)-subtracted fluorescence.

Because of the inhibitory conditions being tested, reactions frequently could not be detected in all replicates. These instances were captured graphically by assigning them to a not-detected (ND) reference line placed at an arbitrary post-assay time of 110 h. For statistical analysis, all times (lag and ND times) were first ranked (PROC RANK) and then analyzed using a generalized linear model based on the gamma distribution (PROC GLIMMIX). Occasional extreme outliers were identified and removed from analyses based on a panel of studentized residual plots. All final models were well fit by the gamma distribution. *Post hoc* analyses consisted of pre-planned comparisons of interest using the modeled least squares means, variation, and the Kenward-Roger method of degrees of freedom estimation for unbalanced data. The family-wise error rate of multiple comparisons was controlled using the adjustment method of Holm (i.e., stepdown Bonferroni). Significance was accepted at a *P* < 0.05.

### Heme-rPrP interaction

Hemin-rPrP interactions were quantified by UV-Vis spectral shift. RT-QuIC buffer without ThT (10 mM NaPO_4_ pH 7.4, 300 mM NaCl, 1 mM EDTA, 0.1 mg/mL rPrP) was spiked with mock sample buffer (1× PBS + 0.1% SDS) or 1× PBS containing hemin at varying concentrations. Due to rPrP precipitation observed upon addition of the SDS+ buffer at 0 µM hemin, the 1× PBS buffer condition was used for binding ratio experiments. Matched hemin-only solutions were also prepared where hemin solutions were spiked into RT-QuIC buffer without ThT or rPrP (10 mM NaPO_4_ pH 7.4, 300 mM NaCl, 1 mM EDTA). Differential spectra were taken, subtracting hemin-only spectra from hemin-rPrP spectra. Evolution of a differential peak at 416 nm and a valley at 385 nm was observed. The difference between these wavelengths was plotted against the hemin:rPrP molar ratio to show dose-dependent evolution of the shift and eventual saturation of rPrP with hemin. Interaction of rPrP with Hb or blood was tested by identical methods to investigate the possibility of heme transfer and/or binding to rPrP from either source.

### Seed exposure to Hb and blood

A 10% brain homogenate from a scrapie-positive sheep was mixed 1:1 with whole blood, Hb solution, or PBS and incubated at 4°C for 24 h or 7 days. Prior to incubation, blood was lysed by sonication for two pulses of 30 seconds each in a water bath sonicator (Qsonica) at 180 W. Hb solution concentration was matched to that of whole blood as measured by ADH assay. Following incubation, mixtures were diluted in SD buffer and used as RT-QuIC seed material as described above.

### rPrP stability in RT-QuIC buffer

RT-QuIC assay buffer (–ThT) containing 0.1 mg/mL rPrP was incubated with mock seed samples containing hemin, Hb, or blood. UV-Vis spectra were then recorded on a CLARIOstar microplate reader (BMG Labtech). Samples were subsequently incubated for 24 h at 42°C. Following incubation, samples were centrifuged at 21,000 × *g* for 10 min, and the supernatant was assayed for rPrP by SDS-PAGE and Coomassie staining.

## RESULTS AND DISCUSSION

### Relative inhibitory effects of blood, hemoglobin, and hemin on prion RT-QuIC

**Fig 1:**
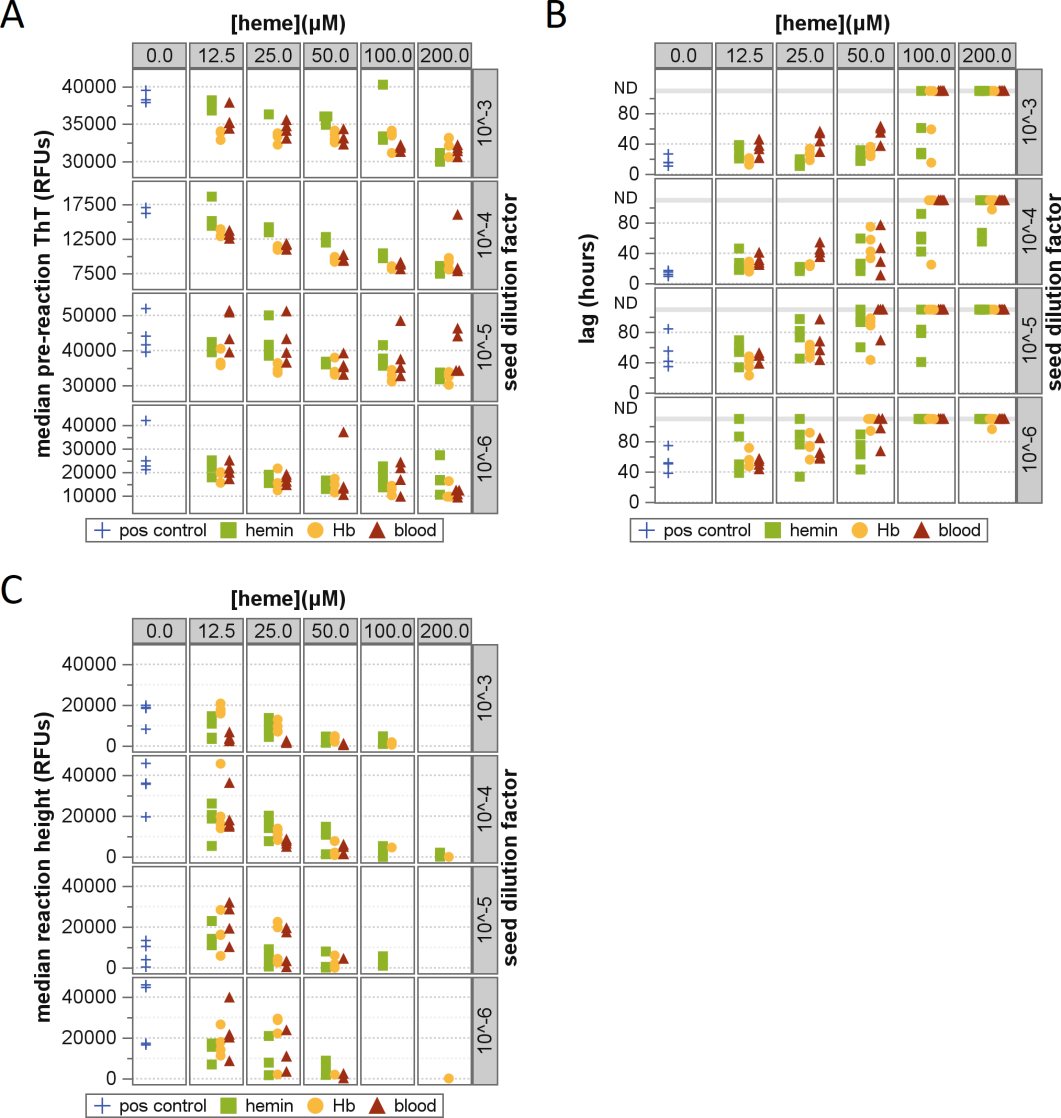
Effects of hemin, Hb, and blood on RT-QuIC detection of prions. Scatter plots depicting (**A**) pre-reaction ThT fluorescence, (**B**) reaction lag times, and (**C**) reaction ThT fluorescence. Reaction outcomes are shown at differing prion seed dilutions (rows), heme concentrations (columns), and heme inhibitor types (markers), with hemin shown as green squares, Hb as yellow circles, whole blood as red triangles, and buffer-only controls as blue crosses. For reaction lag times (**B**), the upper gray reference line denotes wells where no reaction was detected (ND).

To quantify the inhibitory effects of heme, Hb, and whole blood, a matrix of RT-QuIC assay conditions was assembled where scrapie-positive sheep brain homogenates in dilutions ranging from 10^−3^ to 10^−6^ were spiked with heme, Hb, or whole blood at concentrations ranging from 0 to 200 µM (Fig. 1). These concentrations represent the amounts of heme or monomeric Hb present in the seed material prior to RT-QuIC analysis, where 2 µL of seed material is then introduced to 98 µL of reaction buffer. For whole blood, the reported concentrations describe the amount of monomeric Hb the dilution contains.

**Fig 2:**
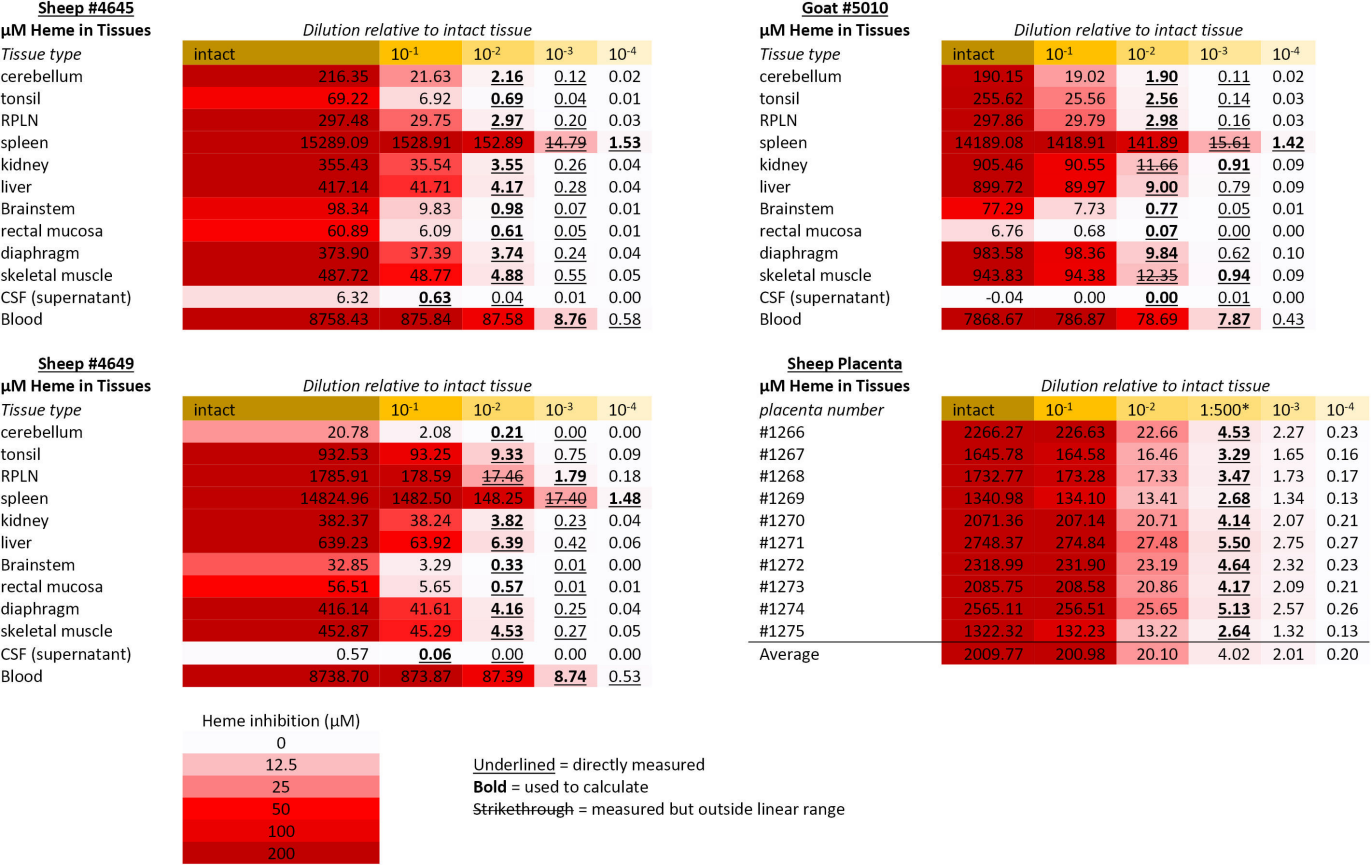
Heme Concentrations and Estimated Inhibition in Small Ruminant Tissues and Homogenate Dilutions. Heme concentrations measured by oxalic acid fluorescence assay. Values for homogenate dilutions directly measured in the assay are underlined. Heme concentrations shown for other homogenate dilutions and intact tissues are calculated from the bolded values. Some measured values fell outside of the standard curve linear range of 0-10 µM and are shown with a strikethrough. For placentas, five biopsies from each placenta were measured at a 1:500 dilution; * mean values for each placenta are shown. Relative tissue homogenate concentrations are highlighted with a yellow color scale. Estimated heme-mediated inhibition levels are highlighted by a red color scale as shown at the bottom.

The obex was chosen as the source of prion seed for several reasons. The obex is the richest source of prions relative to other tissues and, as collected and measured in this study, is very low in natural heme content (≤98 nM for a 10^−3^ dilution; Fig. 2). Use as a simply diluted homogenate also avoids the inherent potential of seed purification methods to perturb the authentic state of prions—whether that be alterations to conformation, solubility, or bound ligands, or to introduce selection bias for subpopulations of prion content.

The addition of heme, Hb, or blood to the seed homogenates resulted in dose-dependent increases in lag times at all seed dilutions (Fig. 1B). At higher inhibitor concentrations, prion detection was completely ablated (Fig. 1B). Of the three inhibitor sources tested, blood appeared to produce the greatest inhibition, followed by Hb, and then heme.

**Fig 3:**
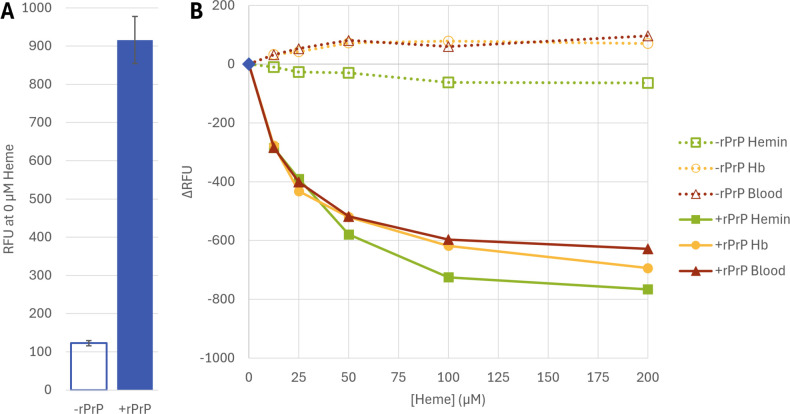
ThT interactions with rPrP and heme-containing inhibitors. Bar chart (**A**) displays mean fluorescence (RFU) for ThT alone or in the presence of rPrP. Error bars denote standard deviation (*n* = 3). Line graph (**B**) shows change in ThT fluorescence (ΔRFU) measured in the presence of 12.5 µM–200 µM hemin, Hb, or whole blood; with or without 0.1 mg/mL rPrP. Values for hemin are denoted by green squares, Hb by yellow circles, and whole blood by red triangles. Mixtures containing rPrP are shown by filled markers and solid lines and reactions without by empty markers and dashed lines.

In addition to the longer lag times and lost detection of seeded reactions, the morphology of the RT-QuIC reaction curves (ThT fluorescence vs time) was changed by the addition of heme-containing inhibitors. In the presence of inhibitors, dose-dependent decreases were observed in the fluorescence signal, both during the pre-reaction period (Fig. 1A) and following amplification (Fig. 1C). Neither hemin nor Hb absorb photons strongly at the excitation or emission wavelengths of ThT (hemin *A*_max_: 385 nm, Hb *A*_max_: 411 nm, ThT_ex_: 450 nm, ThT_em_: 480 nm), and others have demonstrated that the fluorescence of unbound ThT is not impacted by the presence of heme (25). To examine this more closely in our specific reaction conditions, we added the heme-containing inhibitors to RT-QuIC reaction buffer both with and without a standard amount (0.1 mg/mL) of rPrP. Interestingly, while ThT fluorescence is known to increase sharply when bound to amyloid (36, 37), we did observe increased baseline fluorescence in the presence of rPrP (Fig. 3A). The addition of heme-containing inhibitors showed minimal impact on ThT fluorescence in the rPrP-free buffers but did suppress the rPrP-dependent ThT fluorescence increase (Fig. 3B). The contributions of other reaction factors were also analyzed, and the results were summarized as Fig. S1 at https://doi.org/10.15482/USDA.ADC/28836296. One interpretation of the results is that while hemin or Hb do not substantially interact with ThT alone, both molecules appear to interact with rPrP. This interaction may be competitive, i.e., displacing ThT from rPrP, or they may bind concurrently. In the latter scenario, the close proximity of the two bound molecules could allow the heme macrocycle to act as an acceptor for energy transfer, thereby suppressing ThT fluorescence (38).

### Blood/heme does not destroy PrP^Sc^ seeding activity

**Fig 4:**
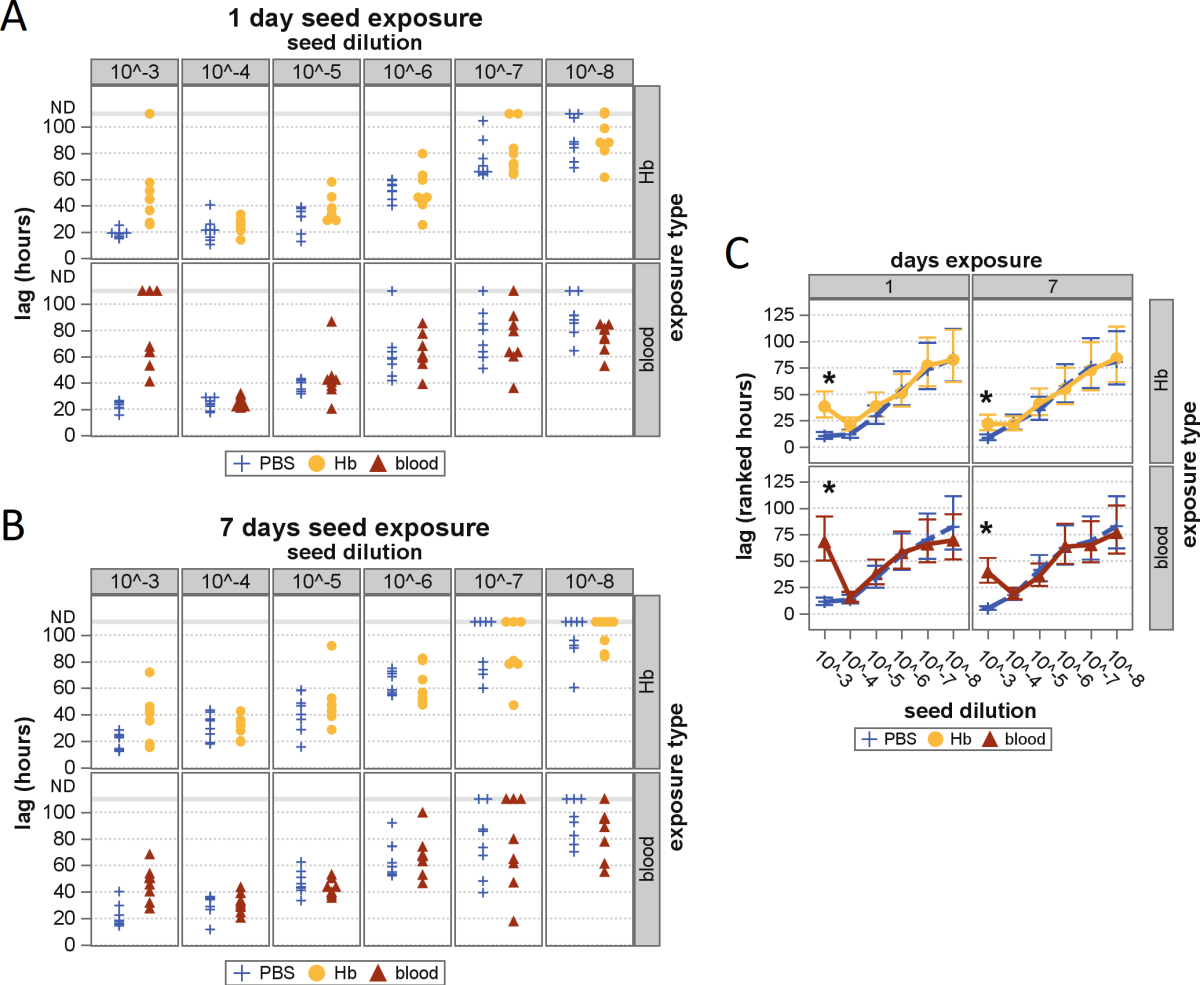
Seeding activity of PrP^Sc^ following exposure to Hb or whole blood. Scatter plots depicting reaction lag times for RT-QuIC following exposure of seed material to Hb (yellow circles) or whole blood (red triangles) for 1 day (**A**) or 7 days (**B**). Controls in each assay were exposed to PBS (blue crosses). Ranked lag times are also plotted against seed dilution factor (**C**) for each condition and its PBS control. Asterisks denote significant differences between ranked lag times from equivalent dilutions of inhibitor-exposed and PBS-exposed samples (all *P_Holm_* ≤0.0006).

Previous research has demonstrated that exposure to heme results in the destruction or restructuring of other pathogenic amyloids (25–28). To test this potential with prions from a natural host, homogenate of a scrapie-positive sheep brain was mixed 1:1 with either lysed whole blood, concentration-matched Hb solution, or 1× PBS and incubated at 4°C for 1 week. These conditions were chosen to mimic those of a severely hemolyzed blood sample or heavily blood-contaminated tissue with subsequent refrigerated storage delays (24 h or 1 week) prior to analysis. The limiting dilution for detection was then determined using serial dilutions of each seed material. Except for the 10^−3^ dilutions in which Hb was still present at >100 µM in the reaction mixture, significant differences in lag time or limits of detection were not observed (Fig. 4). These results demonstrate that exposure to whole blood or hemoglobin in the tissue homogenate does not disrupt the seeding activity of PrP^D^ in the assay.

These encouraging results align with those of another study (39) that examined bloodborne prion detection by a different assay, PMCA. In that study, prion-spiked blood samples did show some changes to detectability in early PMCA rounds but ultimately yielded unhindered limits of detection by serial dilution. As part of its mechanism, the PMCA assay includes serial dilutions between “rounds.” Similar to our conditions at the 10^−3^ dilution, concentrations of blood components in the reaction mixture would be relatively high in these early rounds and be diluted away as the assay progresses, ultimately allowing for detection of the apparently undisrupted prions.

### Inhibitors act on the rPrP assay substrate

**Fig 5:**
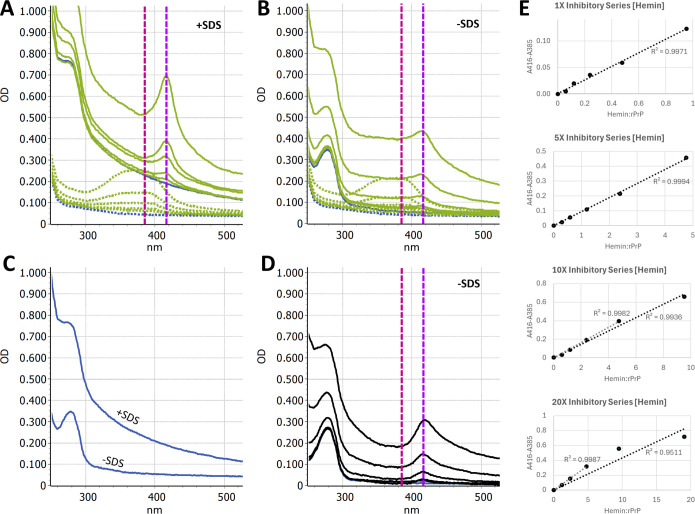
Ratio of hemin:rPrP interaction in RT-QuIC buffer conditions. (**A, B**) UV-Vis absorbance spectra of 0.1 mg/mL rPrP (solid blue), sample buffer only (dashed blue), hemin at 12.5, 25, 50, 100, and 200 µM (dashed green), and 0.1 mg/mL rPrP + hemin (12.5 µM–200 µM) (solid green), shown with SDS (**A**) and without SDS (**B**). (**C**) Spectra of rPrP and 0 µM hemin, with and without SDS. (**D**) Differential spectra of 0.1 mg/mL rPrP + hemin (12.5 µM–200 µM) with hemin spectra subtracted. The differential minimum (385 nm) and maximum (416 nm) are shown with dashed vertical lines. (**E**) Hemin:rPrP binding ratios were tested with 0.1 mg/mL rPrP and multiples of the 12.5 µM–200 µM inhibitory series hemin concentrations. The difference between the differential maximum and minimum (A416–A385) is plotted against the molar ratio of hemin:rPrP. Linear fits and accompanying *R*^2^ values are shown for whole (black dashed line) or partial (gray dashed line) series.

Another fundamental component of amplification assays is the recombinant prion protein substrate. Prior research has shown that rPrP binds to heme in an isoform-specific manner, where the heme:PrP binding ratio is higher for monomeric rPrP than for a misfolded form (23). To confirm similar heme:rPrP binding under RT-QuIC conditions, we spiked RT-QuIC reaction buffer (-ThT) (10 mM NaPO4 pH 7.4, 300 mM NaCl, 1 mM EDTA, 0.1 mg/mL rPrP) with mock seed samples containing from 0 to 200 µM hemin. Binding of rPrP and hemin was observed as a red shift in the maximum absorbance peak of heme (Fig. 5A, B, and D). In addition to the spectral shift, visible turbidity was evident in the spiked samples; this was observed as an overall increase in the UV-Vis spectra absorbance baseline (Fig. 5A B). Interestingly, significant turbidity was also observed in the 0 heme condition, where the reaction buffer was spiked with buffer (1× PBS + 0.1% SDS) alone (Fig. 5A C). To prevent interference from SDS-induced turbidity, heme spiking was also performed using a mock sample buffer of 1× PBS lacking SDS. These conditions did eliminate the turbidity observed at 0 µM hemin (Fig. 5B C) while maintaining the heme binding-induced red shift (Fig. 5B). For this reason, the following experiments examining the binding ratio of hemin:rPrP were performed in the absence of SDS.

To quantify the spectral shift, differential spectra were calculated where the spectra of the hemin solutions alone were subtracted from those of the hemin + rPrP mixtures (Fig. 5D). Evolution of a differential peak at 416 nm and a valley at 385 was observed (Fig. 5D). The difference of these absorbance values was then plotted against the heme:rPrP molar ratio (Fig. 5E). When rPrP was exposed to hemin at the previously tested inhibitory concentrations of 12.5 µM–200 µM, a dose-dependent spectral shift was observed; however, saturation was not reached. Subsequently, hemin was added at 5×, 10×, and 20× the inhibitory concentration series (i.e., 62.5 µM–1,000 µM, 125 µM–2,000 µM, and 150 µM–4,000 µM) to reach saturation. Ultimately, extensive precipitation of rPrP precluded precise quantification of the binding ratio; however, loss of linearity in the red-shift increase was seen beginning at molar ratios greater than ~5:1 hemin:rPrP (Fig. 5E). This broadly agrees with the previously reported 7:1 binding ratio for heme to rPrP (23).

**Fig 6:**
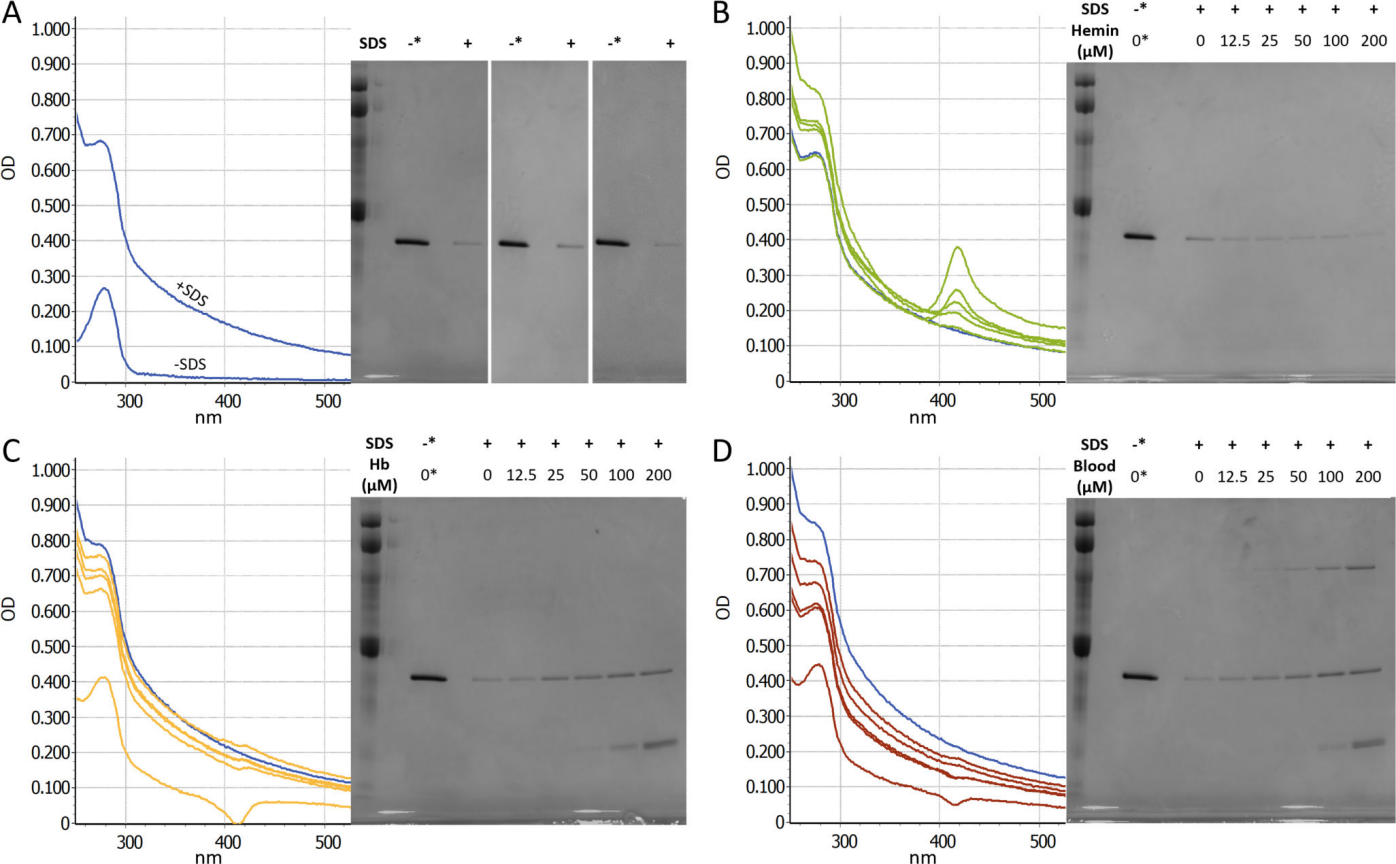
rPrP substrate stability in the presence of SDS and heme-containing inhibitors. Differential UV-Vis absorbance spectra resulting from 0.1 mg/mL rPrP in RT-QuIC buffer (−ThT) exposed to mock seed material containing SDS (0.1% in sample buffer) (**A**) and hemin (**B**), Hb (**C**), or blood (**D**) at 0, 12.5, 25, 50, 100, and 200 µM. In panels B–D, the 0 µM condition is shown in blue. Also shown in (**B**), (**C**), and (**D**) are SDS-PAGE and Coomassie staining of equivalent samples following incubation at 42°C for 24 h and subsequent centrifugation to remove insoluble material. SDS-PAGEs shown in (**A**) are the 0 inhibitor condition from (**B**), (**C**), and (**D**) shown to highlight the similar loss of rPrP in each of these experiments. For Coomassie staining, control samples of 0.1 mg/mL rPrP in RT-QuIC buffer were prepared fresh and not incubated, denoted by an asterisk.

RT-QuIC reaction buffer (−ThT) was also spiked with Hb and whole blood at 12.5 µM–200 µM. While binding of hemin to rPrP displays a strong shift in the differential (heme-subtracted) UV-Vis spectrum prior to incubation (Fig. 6B), initial exposure of rPrP to Hb or whole blood results in a much smaller initial spectral shift (Fig. 6C D). This suggests the majority of heme in these conditions remains bound to Hb or that any transfer of heme to rPrP may occur more gradually over the assay run time. An example figure comparing the differential spectra to the raw and inhibitor-only spectra at the 200 µM conditions can be found in the supporting information (Fig. S2 at https://doi.org/10.15482/USDA.ADC/28836296). To examine the inhibitor impacts at later assay time points under reaction conditions, RT-QuIC reaction buffer was spiked with sample buffer (SDS+) containing hemin, Hb, or whole blood and incubated at 42°C for 24 h. These mixtures were then centrifuged at 21,000 × *g* for 10 min to remove insoluble material, and the resulting supernatants were analyzed by SDS-PAGE and Coomassie staining. In agreement with the initial spectroscopic measurements, even in the absence of heme, a significant portion of the rPrP substrate is lost after 24 h at reaction conditions (Fig. 6A). For the hemin-exposed samples, additional, dose-dependent rPrP loss was observed (Fig. 6B). After 24 h, the Hb or blood spiked buffers unexpectedly showed more rPrP remaining in the buffer than in the 0 heme condition (Fig. 6C and D), indicating that the presence of Hb or blood in the reaction mixture actually stabilizes the solubility of rPrP. These results suggest that both hemin and Hb interact with rPrP in the assay substrate and impact its solubility in the reaction buffer, albeit through different mechanisms.

**Fig 7:**
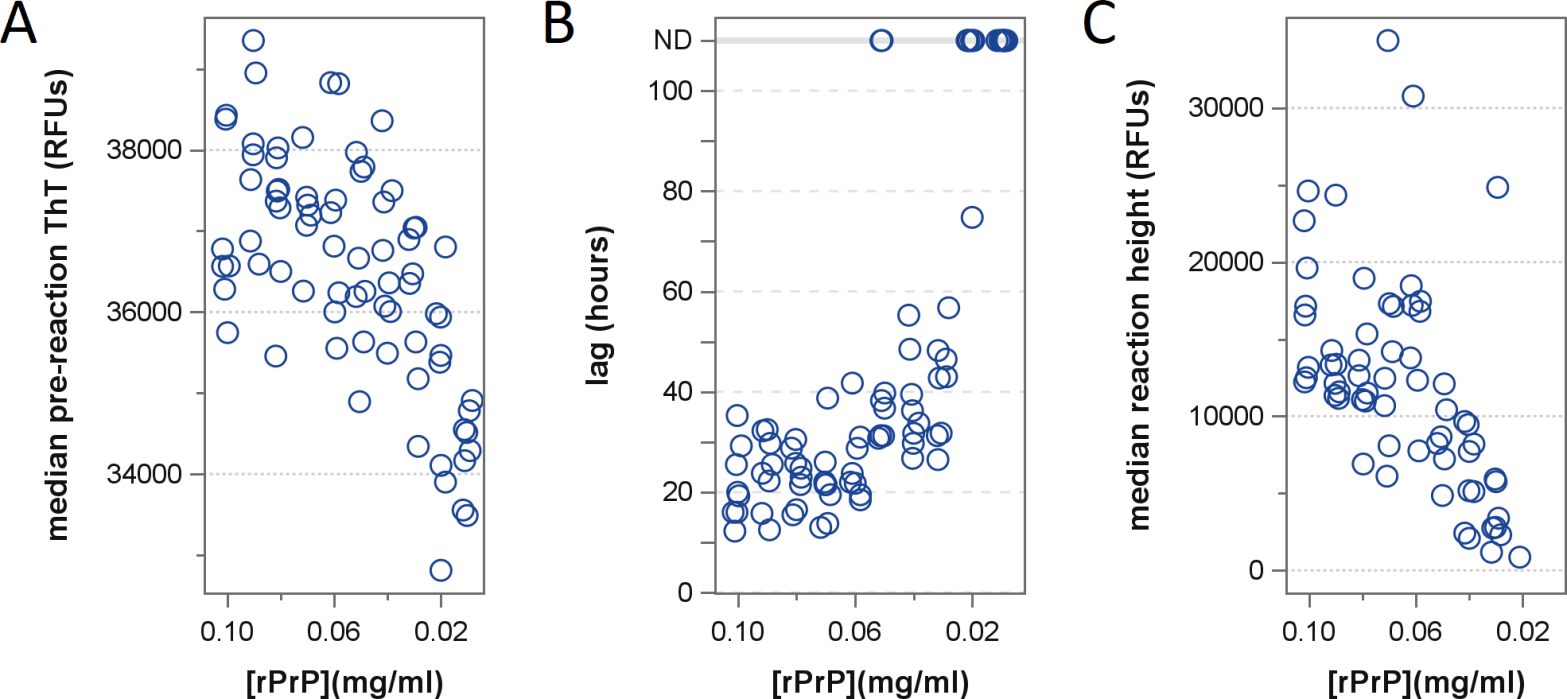
Effects of reduced substrate concentration on RT-QuIC detection of prions. Scatter plots depicting (**A**) pre-reaction ThT fluorescence, (**B**) reaction lag times, and (**C**) reaction ThT fluorescence at reduced rPrP substrate concentrations. Reactions were seeded at a 10^−4^ seed dilution in the absence of heme-containing inhibitors. For reaction lag times (**B**), upper gray reference line denotes wells where no reaction was detected (ND).

In response to the evident changes to rPrP substrate stability following exposure to heme, heme-free RT-QuIC reactions were prepared using a range of substrate concentrations from 0.01 to 0.10 mg/mL (10%–100%) so that the impact of rPrP depletion alone could be assessed. Reactions performed with depleted rPrP, particularly those below 0.05 mg/mL (50%), exhibited longer lag times, lost detections, and diminished fluorescence signals (Fig. 7) similar to the heme-mediated effects on the standard reaction mixture containing 0.1 mg/mL rPrP.

**Fig 8:**
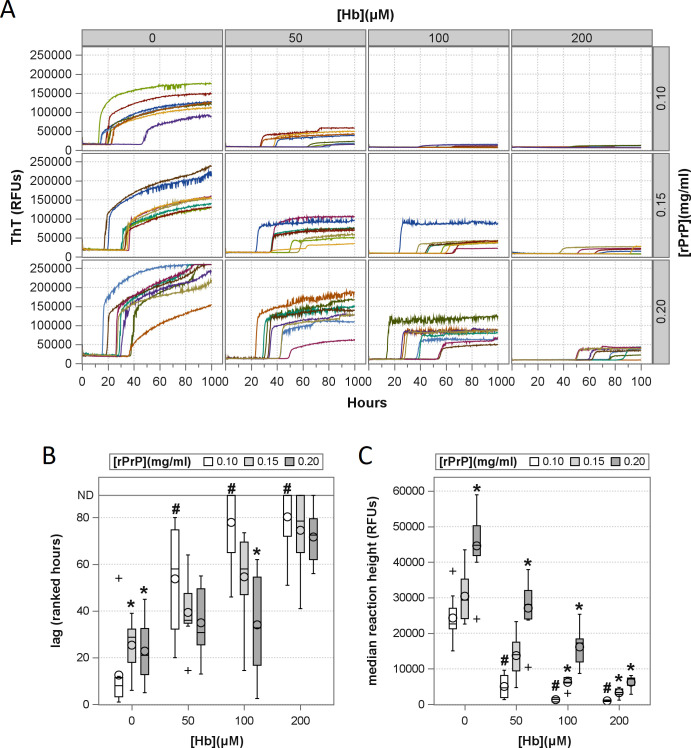
Rescue of RT-QuIC assay inhibition with increased rPrP substrate concentration. (**A**) Raw ThT fluorescence curves for reactions seeded at a 10^−4^ seed dilution in the presence of varying Hb (columns) and increased rPrP substrate (rows) concentrations. Ranked lag times (**B**) and reaction ThT fluorescence (**C**) are shown as bar graphs where # denotes significant differences between Hb-containing and Hb-free reactions at 0.1 mg/mL rPrP (all *P_Holm_* <0.0001), and * denotes significant differences between elevated (0.15, 0.20 mg/mL) and standard (0.1 mg/mL) rPrP concentrations within a given Hb concentration (all *P_Holm_* ≤0.005).

Lastly, a reaction condition matrix was prepared testing reactions containing 0.10, 0.15, and 0.20 mg/mL (100, 150, 200%) rPrP spiked with 0, 50, 100, and 200 µM Hb. As readily observed in Fig. 8A, reactions with elevated rPrP concentrations showed dose-dependent rescue of lag times (Fig. 8B), reaction frequency, and fluorescence intensity (Fig. 8C), albeit with some delay in lag times in the absence of Hb (Fig. 8B, 0 µM Hb). Together, these data demonstrate that interactions with the rPrP substrate are the primary factor by which heme-containing inhibitors disrupt prion detection by RT-QuIC.

As a caveat to mitigating this type of assay inhibition through increased substrate concentration, unseeded (spontaneous) misfolding reactions in the absence of hemoglobin began somewhat earlier at 0.15 mg/mL [rPrP] as compared to that occurring in the standard 0.10 mg/mL [rPrP] buffer (Fig. S3 at https://doi.org/10.15482/USDA.ADC/28836296). This suggests assay cutoff times should be re-evaluated before attempting to apply simple substrate supplementation as a counter to potential heme contamination in a diagnostic setting.

### Heme and protein constituents of Hb each contribute to inhibition of RT-QuIC

**Fig 9:**
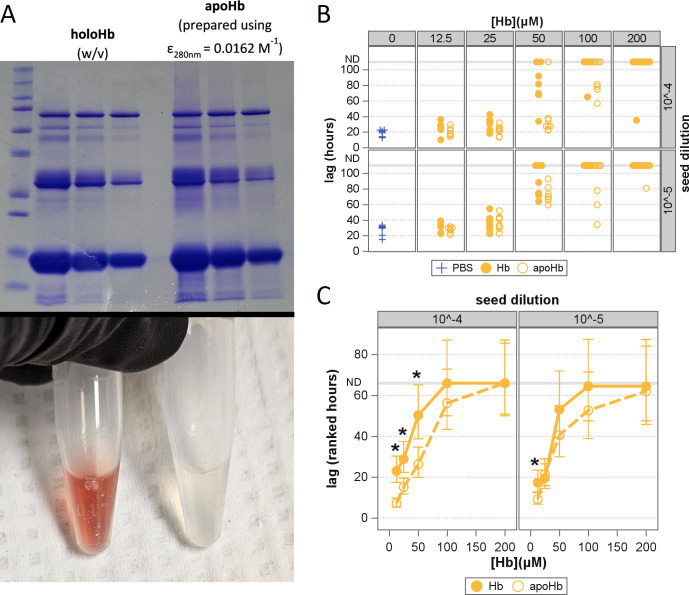
Comparative inhibitory effects of holoHb and apoHb. (**A**) Coomassie-stained SDS-PAGE showing 1:2 dilution series of holoHb (left) and apoHb (right). (**B**) Scatterplot depicting reaction lag times of RT-QuIC reactions at differing seed dilutions (rows), Hb types (markers), and inhibitor concentrations (columns). (**C**) Ranked lag times plotted against inhibitor concentration. Filled yellow circles with solid lines show Hb, and open yellow circles with dashed lines show apoHb. Asterisks denote significant differences between reactions containing equivalent concentrations of Hb or apoHb (all *P_Holm_* ≤0.034).

Given the apparent mechanistic differences between free hemin- and Hb-mediated inhibition, assays were also performed in the presence of apoHb to extricate the respective contributions of the bound heme and globin protein components of Hb. ApoHb was prepared from the holoprotein (Hb) by acidified acetone extraction. Following reconstitution, apoHb concentrations were estimated using the previously published extinction coefficient ε_280nm_ = 0.0162 M^−1^ (29, 31). As apoHb is inherently unstable due to the lack of its native cofactor, absorbance-based protein quantification can be inexact. As a method of secondary confirmation, solutions containing equal amounts of Hb and apoHb (as estimated by 280 nm absorbance) were prepared and analyzed by SDS-PAGE and Coomassie staining. This comparison demonstrated grossly equivalent concentrations between the two forms (Fig. 9A). Though the addition of apoHb did result in assay inhibition relative to 0 µM controls (Fig. 9B), the overall effectiveness in prolonging lag time was generally less for the apoprotein (apoHb) as compared to the holoprotein (Hb) (main effect of Hb type: at 10^−4^, *P* < 0.0001; at 10^−5^, *P* = 0.0316). This was most evident when the reaction was seeded with 10-fold greater PrP^D^, where significant differences between Hb types were detected at 12.5 µM–50 µM Hb (seeded at 10^−4^, *P_Holm_* ≤0.0034), but only at 12.5 µM Hb when seeded at a dilution of 10^−5^ (*P_Holm_* ≤0.0211). These data suggest that both the globin protein component and the bound heme cofactor contribute to the inhibitory effects of Hb on RT-QuIC.

### Heme quantification in diagnostic tissues

To gauge the potential impact of heme/Hb-mediated inhibition on diagnostic efforts using RT-QuIC, various tissues from small ruminants were assayed for total heme concentration by oxalic acid fluorescence assay. The results of these measurements are summarized in Fig. 2, along with calculated extrapolations for various tissue homogenate dilutions. Briefly, while heme concentrations exceed inhibitory levels in nearly all intact tissues, by the time a 10^−3^ dilution was reached, only the blood, spleen, and placenta approached inhibitory levels of heme. However, several tissues maintained micromolar levels of heme if only diluted 10^−2^, which may still be capable of disrupting detection in peripheral tissues or during the earliest stage of infection where tissue samples are more likely to bear low prion titers.

### Conclusions

In this study, we demonstrate that each of heme, Hb, and whole blood inhibits the RT-QuIC assay. Delayed lag times were observed with seed material samples containing as low as 12.5 µM heme and, in the case of lower prion-titer samples, a substantial loss of replicate detections was observed at concentrations as low as 50 µM for Hb or whole blood. Heme itself, the globin component of Hb, and the remaining constituents of whole blood are each contributing factors to the observed inhibition. When prion-containing samples were incubated in the presence of Hb or whole blood prior to being assayed, seeding activity was not lost, suggesting that inhibition occurs at the level of the assay itself. Hemin was confirmed to be able to bind rPrP in RT-QuIC reaction buffer, and both free hemin and Hb induced solubility changes in the protein substrate. When heme-free reactions were performed with limited quantities of substrate, outcomes mirrored those of reactions inhibited by heme, Hb, or blood. Finally, increasing the starting rPrP concentration was able to rescue Hb-inhibited prion detection, reinforcing the conclusion that inhibition from Hb- or blood-containing samples is a result of depletion of available rPrP substrate in the assay mixture.

**Fig 10:**
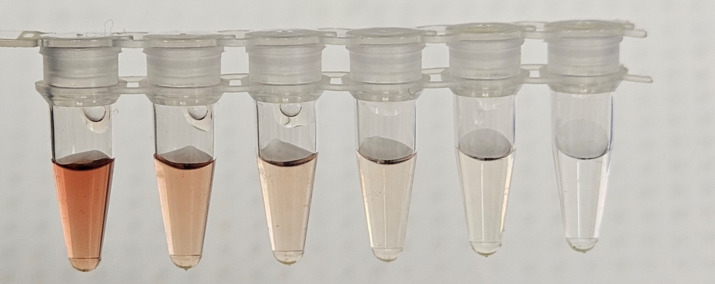
Visual appearance of inhibitory Hb concentrations. Hb in sample buffer at 200, 100, 50, 25, 12.5, and 0 µM.

When heme was measured in small ruminant tissues, levels were, not unexpectedly, shown to vary between tissue types. Of interest, nearly all types of intact tissue contain >200 µM heme; however, given our data suggesting pre-reaction exposure does not degrade PrP^D^ seeding activity, the concentrations at the actual testing dilutions are more relevant. At the most commonly tested tissue dilution, 10^−3^, only the blood, spleen, and placenta still contained millimolar quantities, and even these tissues would be at the low end of the inhibitory range. It should be noted that the tissues in this study were collected from freshly euthanized animals which were then exsanguinated and necropsied in a timely manner. It is therefore possible that field-collected samples may have a considerably greater degree of blood contamination or hemolysis. Even so, given the results from whole blood and splenic tissue, it remains unlikely that heme-mediated inhibition would be more than a minimal factor at a 10^−3^ dilution or greater. While these levels of sample dilution are common practice, they are inherently limiting to the theoretical sensitivity of the assay. In recent years, a variety of efforts have been made to enrich or purify prions from tissues (10, 40–42), some of which are only semi-selective for PrP^D^ and may result in increased inhibitor concentrations along with the enriched prions. Other strategies aim to allow for testing of less diluted samples (43, 44). In these cases, it is possible that heme or Hb levels may again be present at inhibitory levels. Somewhat conveniently, the ability to see the color of a solution of aqueous Hb with the naked eye roughly coincides with the inhibitory ranges described in this study (Fig. 10). As a broad observation, if the final seed material to be introduced to the RT-QuIC assay is visibly red/pink/brown colored, Hb-mediated inhibition may influence the reaction lag times, fluorescence signal maxima, and frequency of detection, and thus should be considered when interpreting results.
